# A descriptive study of trauma cases encountered in the Grand M’Bour Hospital Emergency Department in Senegal

**DOI:** 10.11604/pamj.2019.32.9.14550

**Published:** 2019-01-07

**Authors:** Kenneth John, Farba Faye, Rhonda Belue

**Affiliations:** 1 Pennsylvania State University College of Medicine, Hershey, PA, USA; 2 Hopital de Grand Mbour, Mbour, Senegal; 3 Department of Health Management and Policy, St Louis University, St Louis, MO, USA

**Keywords:** Trauma, Senegal, mechanisms of injury, epidemiology

## Abstract

**Introduction:**

This study analyzed the trends of trauma cases that presented to the Emergency Department (ED) in the Grand M'Bour Hospital. We examined demographics of patients, mechanisms of trauma and types of injuries that result and times from injury to arrival.

**Methods:**

This was a descriptive study using prospective ED trauma cases. Patients were selected for the study if their chief complaint was related to a traumatic injury. A trauma flow sheet was developed to obtain information. Data was collected from 6/22/16-7/13/16, with 105 cases recorded. Abstracted data included date, time of arrival, time of injury, age, gender, mechanism of injury, injury sustained and disposition.

**Results:**

Patients presented to the ED for 13 different trauma-related reasons. 71% of the patients encountered had a mechanism of injury related to falls or motor vehicle accidents. The majority of patients who suffered from a fall-or motor vehicle-related injury were children, with ages 0-10 representing 31% and ages 11-20 representing 14% of the total patients. While 29% of patients were seen within 1 hour of the time of their injury, 10% of the patients were not seen until days after their injury.

**Conclusion:**

We report that traumatic injuries are most commonly a result of fall-related and vehicle-related accidents. Children under the age of 20 years old are a vulnerable population for traumatic injuries. We observed that many patients were unable to seek care within a day of their injury. This was concerning that proper emergency transportation was not available, leading to potential complications or improper healing of injuries. Knowing these trends, an ED can be better prepared to treat these patients.

## Introduction

Injuries have become a burgeoning epidemic in developing countries, causing more than 5 million deaths each year, roughly equal to the number of deaths from HIV, malaria and tuberculosis combined [1]. Because of the well-recognized burden of infectious disease and malnutrition, the effects of trauma and injuries on premature mortality and long-term disability are often overlooked [2]. Low-and middle-income countries are home to more than 90% of injury deaths worldwide, with motor vehicle accidents heavily contributing to that number. Africa's road traffic injury mortality rate, 32.2 per 100,000, doubles that of America's rate [3]. With economic development in low-income countries, we see an increase in the number of vehicles and an associated rise in traffic-related crashes, injuries and deaths [1]. Due to the economic growth in low-and middle-income countries, motor vehicle accidents are projected to increase from 1.2 million to 1.9 million from 2002 to 2030 [4]. Emergency Medicine is a relatively new field in the Sub-Saharan region of Africa. A study on hospitals in Ghana, Kenya, Rwanda, Tanzania and Uganda showed that none of the hospitals surveyed had enough infrastructure to follow the minimum standards and practices that the World Health Organization (WHO) has deemed essential for the provision of emergency and surgical care. In fact, only 19-50% of hospitals had the capability to provide 24-hour emergency care [5]. In Ghana, the trauma mortality rate is estimated to be 80-100 per 100,000 patients each year-approximately 50% greater than the trauma mortality rate in a developed country [6]. The problem lies beyond the resources available in African hospitals. A preponderance of evidence supports that traumatic deaths happen in the prehospital setting more frequently in developing countries in sub-Saharan Africa compared to developed countries [6]. This shines light on the potential in these countries to develop prehospital care plans, such as an Emergency Medical Services (EMS) system. The time between an injury and when the patient arrived to the Emergency Department can vary significantly. This stems from problems such as unpaved roads and inequalities between rural and urban communities for access to an emergency response system [2].

Evidence supports that providing emergency transport can save lives by stabilizing and treating the patient earlier. A study in Sierra Leone demonstrated that an investment in an emergency vehicle and an improved communication system led to a doubling of the utilization of emergency obstetric services, as well as a 50% reduction of obstetric-case fatalities [7]. EMS systems are very costly and take time to fully develop. Therefore, countries have analyzed and found ways to build on upon their current system. For example, a study in Ghana showed that commercial vehicles-taxis, buses and trucks-were the most common modes of transportation of patients to the Emergency Department. Knowing this, commercial vehicles became the building block to further development of the Ghana EMS system [6]. Taking these strides toward improving prehospital care can allow patients to seek medical care more quickly and reduce the mortality rate from trauma. A growing emphasis also focuses on injury prevention in low-to middle-income countries to decrease the burden of injury on mortality. Although traumatic injuries have a high correlation with premature mortality and disability, certain patient populations seem to be more affected. Road traffic injuries make up the leading causes of death and disability in the 5 to 44 year age group. Gender disparities exist as well, showing that road traffic injuries are the leading cause of death in males ages 15 to 44 years old, whereas suicide is the leading cause among females [2]. Populations at greatest risk for vehicle-related injuries include elderly, children pedestrians, cyclists, and riders of two-wheeled motor vehicles [2]. A disparity of injuries also depends on where patients live geographically, with rural areas experiencing more long-term disability from extremity injuries and urban areas encountering more head and spinal cord injuries [8]. By understanding who is affected most by traumatic injuries, communities can better target them for prevention practices. Examining injury duration prior to initiation of medical care can allow communities to delve into why certain populations or injuries may take longer to seek medical attention. This article will explore the demographics of trauma victims at an urban hospital in Senegal, the time duration from injury to arrival in the ED, the mechanisms of injury common to the region and injury types most commonly reported.

## Methods

**Objective:** The objective of this study was to examine the trends of trauma cases that presented to the Emergency Department in the Grand M'Bour Hospital. Specifically, we examined the mechanisms of trauma and the types of injuries that resulted. In addition, we assessed the transport times from injury to arrival in the ED.

**Setting:** This study took place in the Emergency Department at the Grand M'Bour hospital, a regional medical facility located in the Thies region of Senegal. M'Bour is approximately 42km South of the capital of Senegal, Dakar. The hospital serves a catchment area of approximately 500,000 people, the majority of whom are underserved and uninsured. It has 116 inpatient beds and 19 maternity beds. As of the writing of this manuscript, the hospital is operated by 19 doctors and nearly 140 other staff members. The Emergency Department has 11 beds and is staffed by two Attendings, approximately five medical students, and five nurses. The ED treats about 1,500 patients per month. The Grand M'Bour Hospital has ambulance services; however, ambulances arriving at the facility typically provide hospital-to-hospital transportation.

**Study design:** This was a descriptive study using prospective ED cases that presented to the Grand M'Bour Hospital. Patients were selected for the study if their chief complaint or reason for visit was related to a traumatic injury. A trauma flow sheet was developed to collect information about each case presenting between 6/21/16 and 7/13/16. The Attendings and medical students filled out the trauma flow sheets. They were trained by Dr. Farba Faye, the Director of the ED, to identify patients who would qualify for the study and what information needed to be collected. The sheets were collected from 6/21-7/13, with 105 cases recorded. The sheets were then translated from French to English with the assistance of Dr. Faye. Data was entered into an Excel spreadsheet and the abstracted data included date, time of arrival, time of injury, age, gender, mechanism of injury, injury sustained and disposition.

**Ethics:** The lead authors' Institutional Review Board/ Ethics Committee (IRB) and at Pennsylvania State University approved this study. The partnering hospital does not have an IRB or ethnics committee. Collaborators at the local partnering hospital relied on the lead authors institution's IRB as the ethics committee of record, were aware of the IRB process and provided a letter of support for participating in the current study as part of the ethics process. Our trauma flow sheets were collected from staff and stored in the director's locked office for further reference. Patient privacy was ensured by de-identifying each patient when their information was added to the Excel spreadsheet. No names, date of births, or medical record numbers were added to the spreadsheet. Each patient was identified on the Excel spreadsheet as a number, 1 through 105.

### Measures

**Demographics:** Age (in years) and patient gender (male or female) were obtained from most case-in 4 cases, the ages were not documented on the trauma flow sheet.

**Transport time:** The time of arrival in the emergency department was recorded for each patient as he or she arrived. The time of the injury was determined by asking the patient or family members. Transport time (in hours) was then calculated by taking the difference in time from the incident of the injury to when the patient arrived at the hospital. If the injury occurred more than a day before the patient's arrival and the exact time of injury was unknown, each day until his or her arrival was estimated as 24 hours. All transport times were rounded to the nearest hour with the exception of any time under 1 hour represented as a 0 on the Excel spreadsheet.

**Mechanisms of injury:** These mechanisms were determined by the history of the patient's injury provided by the patient or a family member. This study included 13 different mechanisms that were observed-motor vehicle crash (MVC), fall, child abuse, unarmed assault, armed assault, work accident, domestic accident, pedestrian vehicle crash (PVC), animal-related injury, burn, sport-related injury, boating accident and drowning.

**Injuries sustained:** These injuries were determined by both the physical exam of the patient and radiographic studies including x-rays and CT scans when applicable. Injuries were first divided into two categories-fractures or injuries excluding fractures. If the patient experienced a fracture then the bone injured was identified and recorded. If no fracture was present then the next most critical complaint or injury for the patient was recorded-bruise, laceration, dislocation, swelling, ecchymosis, sprain, burn, tenderness, compression, abrasions-as well as the location of the injury. These were then categorized using the International Statistical Classification of Diseases and Related Health Problems (ICD-10), developed by the WHO.

**Patient disposition:** Disposition was recorded based upon the final outcome of the patient's treatment in the ER. The dispositions encountered during this study include discharge, admission to surgery service and follow-up with specialist such as orthopedics, otolaryngology, dental care, or neurosurgery. Mortality was also recorded if that patient died while in the emergency department.

**Figure 1:**
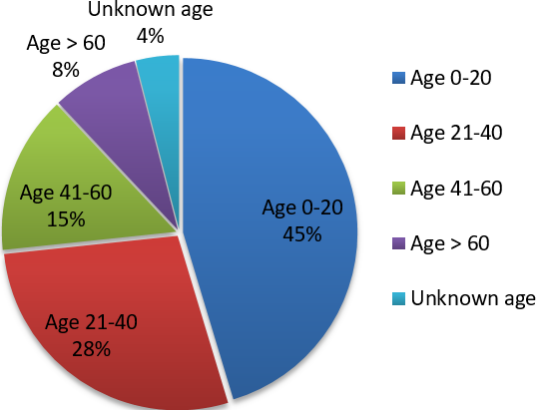
Age distribution of the top 3 mechanisms of injury combined: falls, motor vehicle accidents and pedestrian vehicle accidents

**Analytic approach:** All tables and charts presented in this paper were created using Microsoft Excel Spreadsheet. Table 1 was created by analyzing which mechanisms of injury were most commonly seen in the ED. Table 2 was created by analyzing which injuries were most commonly sustained. The category "Other" includes multiple superficial injuries of hip and thigh, contusion of knee, multiple superficial injuries, contusion of ankle, sprain/strain of other and unspecified part of knee, fracture of upper end of ulna, open wound of abdominal wall, fracture of metatarsal, contusion of other parts of wrist and hand, open wound of wrist and hangs, multiple superficial injuries of ankle and foot, open wound of elbow, fracture of rib, multiple open wounds unspecified, open wound of lower leg, dislocation of shoulder joint, contusion of shoulder or upper arm, multiple open wounds to the abdomen, lower back, and pelvis, multiple fractures unspecified, fracture of patella, fracture of other parts of lower leg, contusion of eyelid and periocular area, contusion of toe, open wound of forearm, open wound of upper arm, open wound of thigh, open wound of front wall of thorax, contusion of lower leg, open wound of foot, superficial injury of hip and thigh, burn to hip and lower limb, fracture of toe, fracture of finger, drowning, and unknown injuries. When calculating the average duration of injury prior to arrival to the hospital, any injury duration greater than 3 days was rounded to 72 hours to decrease the prevalence of outliers. Figure 1 was created by calculating the percentage of patients in various age decades affected by the top three mechanisms seen in this study-falls, motor vehicle accidents and pedestrian vehicle accidents. Table 1, Table 2 and Figure 1 are geared to describing what demographics of the Grand M'Bour community are affected by trauma, what the common causes of trauma are and what types of injuries are occurring in the community.

**Table 1:** Mechanisms of injury

Mechanism of Injury	*n*	Percent
Fall	40	38.1
Motor Vehicle Accident	25	23.8
Pedestrian Vehicle Accident	10	9.5
Armed assault	10	9.5
Unarmed assault	4	3.8
Sport-related injury	4	3.8
Domestic Incident	3	2.9
Work Incident	2	1.9
Boating Accident	2	1.9
Child Abuse	1	1
Animal-related Injury	1	1
Burn	1	1
Drowning	1	1
Unknown Mechanism	1	1
**Total**	**105**	

**Table 2:** Most frequent injuries sustained and average duration of time to treatment

Top 11 Injuries Sustained (categorized by ICD-10 Classification)	*n*	*Average Duration of Injury Prior to Treatment (hours)*
Fracture of Shaft of Tibia	9	13.7
Open Wound of Head	8	1.4
Fractures of Shafts of both Ulna and Radius	7	18.7
Fracture of Shaft of Humerus	7	12.7
Multiple Superficial Injuries of the Head	6	2.2
Other Specified Injuries Involving Multiple Body Parts	6	15.7
Fracture of Shaft of Femur	4	23.5
Fracture of other and unspecified parts of lumbar spine and pelvis	3	31.7
Fracture of Clavicle	3	1.5
Fracture of other Skull and Facial Bones	3	0.7
Sprain or Strain of Ankle	3	2.0
Other	46	17.0

**Figure 2:**
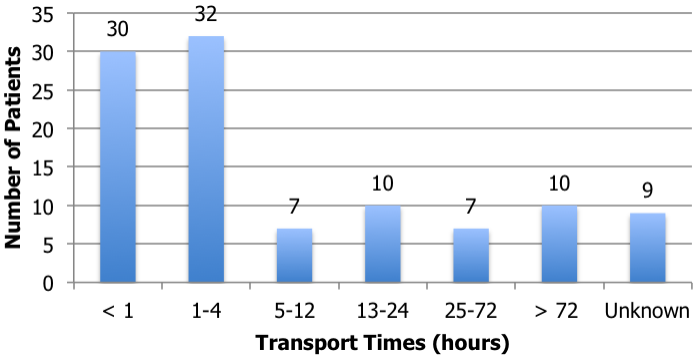
Prehospital transport times

Figure 2 was created by analyzing the number of patients who had transport times <1 hour, spanning 1-4 hours, 5-12 hours, 13-24 hours, 25-72, or greater than 72 hours. Table 3 was created by analyzing what types of injuries were sustained by patients who did not receive medical attention within 24 hours of their injuries. The ICD-10 codes for injuries sustained were categorized into broader categories to simplify the data. "Burn to Lower Extremities" includes burn to hip and lower extremity. "Contusion of Face" includes contusion of eyelid and periocular area. "Contusion of Extremities" includes contusion of ankle, knee, other parts of wrist and hand, shoulder or upper arm and toe. "Joint Dislocation" includes dislocation of shoulder. "Fracture of Head, Neck, or Spine" includes fracture of neck (part unspecified), other and unspecified parts of lumbar spine and other skull and facial bones. "Fracture of Extremities" includes fracture of finger, metatarsal, other parts of lower leg, patella, femur, humerus, tibia, toe, and upper end of ulna. "Fracture of Torso" includes fracture of ribs and clavicle. "Multiple Fractures" includes fractures of shafts of both ulna and radius and multiple fractures unspecified. "Superficial Injuries" includes superficial injuries to hip and thigh. "Multiple Superficial Injuries" includes multiple superficial injuries of ankle and foot, hip or thigh, the head, or parts unspecified. "Open Wound to Head or Neck" includes open wounds to the head. "Open Wound to Extremities" includes open wounds to the forearm, lower leg, elbow, thigh, upper arm, and wrist and hands. "Open Wound to the Torso" includes open wound of abdominal wall and front wall of thorax. "Sprain or Strain of Joint" includes sprains or strains of ankle or other and unspecified parts. Figure 2 and Table 3 were created to analyze how long patients endured certain traumatic injuries before being able or willing to seek medical care.

**Table 3:** Injuries with a duration greater than 24 hours prior to arrival to the ED

Injuries sustained	25-72 hours	> 72 hours
Contusion of Extremities	0	3
Joint Dislocation	0	1
Fracture of Head, Neck, or Spine	0	1
Fracture of Extremities	1	3
Multiple Fractures	2	0
Open Wound to Extremities	3	0
Multiple Open Wounds	0	1
Other Specified Injuries Involving Multiple Body Parts	0	1
Sprain or Strain of Joint	1	0
***Total***	***7***	***10***

## Results

Over the course of three weeks, a total of 105 patients who were victims of traumatic injuries were identified. Of these patients, we were unable to determine one patient's mechanisms of injury, one patient's subsequent injury, three patients' ages, and nine patients' transport times. These omissions were a result of either the critical nature of the patient's injury and lack of time to collect the data or the patient was unable to provide the information due to incapacitation or lack of knowledge. Patients presented to the ED for 13 different trauma-related reasons (Table 1). Fall-related injuries and motor vehicle-related injuries comprise the majority of reasons patients visit the ED (71.3%). For the purpose of our results, we divided motor vehicle-related injuries into two groups: motor vehicle accidents and pedestrian vehicle accidents. The patient in motor vehicle accidents was either the driver or a passenger. Assaults were also a relatively common mechanism of injury (13.3%). The rest of the mechanisms were only seen four times or fewer. Figure 1 represents the age distribution of patients who suffered injuries from falls, motor vehicle accidents, and pedestrian vehicle accidents. Almost half of the patients are children, with patients 20 years old and younger representing 45% of the total patients. The second highest age group is ages 21-40 with 28%. Then, as the ages of the patients increase, the number of injured patients declines. There were also many different unintentional injuries sustained by patients. Of them, fractures of long bones and head injuries are most commonly diagnosed (Table 2). The injuries, diagnosed by physical exam and radiographic evidence, were categorized using the International Classification of Diseases (ICD-10) codes. Most patients presenting to the ED came in by private vehicle, public transportation, or walking. These modes of transportation only lengthen the critical time that patients endure before they are treated. 30 patients (29%) were seen within 1 hour of the time of their injury and 62 patients (59%) were seen within four hours. Still, many other patients were not seen until days after their injury (Figure 2). Injuries from which patients waited 72 hours to arrive to the ED included ulnar and radius fractures, lumbar spine/pelvis fracture, femur fracture, tibia fracture, shoulder dislocation, patella fracture (Table 3). Some of these injuries become life threatening and potentially irreversible as time elapses without treatment.

## Discussion

The Emergency Department (ED) plays a pivotal role in both the hospital and the public health of the community. Emergency medical care has three main components: care in the community, care during transportation and care on arrival at the receiving facility [9]. The purpose of this study was to investigate the demographics of trauma patients, their mechanisms of injuries, the injuries they sustained and their transport times to better understand the trauma cases that the Grand M'Bour Hospital Emergency Department was facing. Our results greatly agree with current literature that road-traffic injuries are a major component of trauma cases in Senegal and similar countries and are especially common in people younger than 20 years old [2, 3]. Communities can use this information to help decrease the number of traumas related to motor-vehicle accidents by creating safer roads, imposing more severe sanctions for unsafe driving practices, and educating parents and children about safety practices as both pedestrians and drivers. While Emergency Medicine (EM) is still in its infancy in Africa, there is a great need for expansion of EM education for physicians and accessibility of care for patients. Africa's first EM residency program began in South Africa in 2004 [3]. Principles and concepts of EM are found to be underrepresented in or absent from their curriculum [10]. There is a push for the globalization of EM by international organizations, such as the International Federation for Emergency Medicine and African Federation for Emergency Medicine, to expand the training opportunities and support for EM residency programs [3]. In 2009, the Ghana College of Physicians and Surgeons (GCPS) accepted a proposal to create a three-year Emergency Medicine residency program [11]. Although there is growing interest in EM training, the GCPS program is limited by funding availability to increase their class size [11]. The role of emergency nursing is also evolving across Africa in areas such as Rwanda, Malawi and South Africa [11]. In South Africa specifically, there are private and public nursing schools that offer advanced diplomas or a master's degree in emergency nursing [12]. However, currently there is no professional board that exists to define the scope of practice for an emergency trained nurse, comparable to the Emergency Nursing Association in the United States of America [12]. As educational models for EM training arise and become established in low-and middle-income countries in Western and Southern Africa, surrounding countries can use them for guidance and resources to implement EM training within their own regional medical facilities. Our results showed a variety of long-bone fractures and head injuries as the most frequent injuries sustained. This data helps to determine specific areas of medicine and injuries that physicians can focus on in Emergency Care education. By recognizing common injuries and pathologies, physicians can better prepare themselves to handle patients presenting with these complaints and become more fluent in treatment and management to decrease long-lasting adverse effects. Effective and sustained EM programs and practices can have a beneficial impact on the public heath of countries at all levels of socioeconomic development [10]. While the majority of the patients observed in this study were seen in the ED within four hours of their injury, an alarming number of patients still waited longer than four hours to arrive to the ED. This delay has potentially fatal consequences. For example, prompt surgical treatment within four hours of a subdural hematomas, an injury commonly associated with road traffic injuries and falls, had a 60-70% functional recovery rate, compared to only 10% in patients who were operated on greater than four hours from their injury [13].

Table 3 illustrates that some of the most critical injuries that presented to the ED, such as fractures to extremities or the head and neck region and joint dislocations, were untreated for longer than 24 hours. These injuries can become life threatening as the duration of time before medical treatment increases. Many factors play a role into this delay of care, mainly: a lack of or inaccessible EMS services based on geographical location, unpaved roads, a lack of regional hospitals with adequate emergency care resources, and use of traditional healers [2, 14]. In 2004, the government in Ghana created the National Ambulance Service (NAS), with Regional Medical Coordinators responsible for managing EMS in various areas of the country [11]. In 2014, Ghana's NAS reported only 8,000 patient transports annually, with 70% of those being inter-facility transfers. These statistics are similar to what we encountered in the Grand M'Bour ED, with the majority of ambulance patients arriving or leaving as result of inter-facility transfers. The Ghana NAS concludes that their low number of annual ambulance transports is a result of poor citizen knowledge of NAS, limited infrastructure and resources and long mission times [11]. When accessibility to hospitals or medications is inadequate, for either financial or geographical reasons, many people will turn to a traditional healer to cure their medical ailments. According to the WHO, a traditional healer is "a person who is recognized by the community where he or she lives as someone competent to provide health care by using plant, animal and mineral substances and other methods based on social, cultural and religion practices" [14]. Traditional healers have a place in medicine, but certain time-sensitive illnesses require prompt EM care. We can work to reduce this delay in accessing care through public education regarding how and when to seek EM care [10]. Benefits also exist to teaching community volunteers simple but important interventions such as establishing and maintaining a patent airway, controlling external bleeding and immobilizing fractures with available resources [9]. With these various factors that impact a delay of treatment for patients with emergent injuries, underdeveloped countries can approach this problem from different avenues. It is wise and may be most beneficial where resources are scarce, to first analyze an existing system and identify areas that have the most potential for growth and that will have the most impact to decrease the transport times for patients [6]. A task like this will need collaboration from various groups of people in medicine, government, and the community to effectively help decrease the mortality rate from traumatic injuries. Additionally, low-and middle-income community hospitals should implement a systematic triage system to insure their limited human and physical emergency resource capacity is being utilized for appropriate patients, which can help decrease the time a critical patient is waiting to be treated by provider. The South African Triage Scale (SATS), developed in 2004, has proven to be a reliable and validated tool that has been implemented in various western African countries, including Ghana [15]. The SATS divides patients into five different urgency categories based on a brief clinical assessment of a patient, with each category indicating how quickly a physician should see them [16]. Implementation of SATS, along with training in EM, has shown to reduce wasting of medical resources and reduce morbidity and mortality in patients [15]. This study was not without limitations. Our study only represents a single medical center in Senegal and may not be representative of the entire country. Longitudinal studies, improved record keeping to facilitate both prospective and retrospective studies over longer periods, as well as large multi-center studies are needed to better ascertain the reality of trauma and emergency care in Senegal.

## Conclusion

Emergency care takes place in the community, transportation and hospital setting. Community education and improvements in EMS can help to decrease transportation times to the ED and decrease overall morbidity and mortality from traumatic injuries. As the burden of traumatic and emergent injuries grows in Senegal and other low-to middle-income countries, it is imperative that health care providers who are tasked with working in Emergency Departments seek out training in emergency management and care. It is also important to gain an understanding of the injuries and diseases that plague their surrounding region in order to be prepared and provide the highest level of care for patients.

### What is known about this topic

- The road traffic injury mortality rate in Senegal (27.3 per 100,000) is more than twice America's road traffic injury mortality rate (10.6 per 100,000);
- Traumatic deaths happen in the prehospital setting more frequently in developing countries in sub-Saharan Africa compared to developed countries;
- Providing emergency transportation can save lives by stabilizing and treating the patient earlier.

### What this study adds

- The most common mechanisms of injury encountered were fall-related and motor vehicle related;
- Pediatric patients, ages 0-20 years old, were the most commonly affected age group;
- Most trauma patients arrived to the ED via private vehicle, public transportation, or walking, which all lengthen the transport time to the hospital.

## Competing interests

The authors declare no competing interests.
